# Release of pseudosyndactyly in recessive dystrophic epidermolysis bullosa using a dermal regeneration template glove: the Foggia experience

**DOI:** 10.1186/s13023-021-01697-5

**Published:** 2021-01-28

**Authors:** Fedele Lembo, Domenico Parisi, Liberato Roberto Cecchino, Francesco Ciancio, Alessandro Innocenti, Aurelio Portincasa

**Affiliations:** 1 Department of Plastic and Reconstructive Surgery, Ospedali Riuniti University of Foggia, Viale Pinto1, 71100 Foggia, Italy; 2 Plastic and Reconstructive Microsurgery, Careggi Universital Hospital, Florence, Italy

**Keywords:** Epidermolysis bullosa, Pseudosyndactyly, Hand, Dermal regeneration template

## Abstract

**Background:**

Epidermolysis bullosa (EB) comprises a heterogeneous group of rare genetic diseases associated with skin blistering caused by minimal trauma. A major and common EB subtype, recessive dystrophic EB (RDEB), is characterized by altered wound healing, inflammatory dysbalance and fibrotic changes associated with reduced to absent collagen VII. Because of its exposed position and its continued use in daily activities, the hand is constantly at risk of microtrauma and is therefore one of the organs most affected by the disease with highly disabling deformities that represent a challenging field in hand surgery practice.

**Methods:**

The authors present their experience in the microsurgical treatment of pseudosyndactylies comparing the classic dressing with vaseline gauze with an innovative “glove protocol” using Integra^®^ dermal regeneration template. The endpoints analyzed were: healing times, hospital stay time, discomfort for the patient, free-recurrence interval, follow-up range and major complications.

**Results:**

A total of 34 procedures were performed on 24 RDEB patients with hand deformities. Compared with the dressing with vaseline gauze, microsurgery followed by application of dermal regeneration template gloves allowed a significant reduction of hospital stay, healing time, and dressing pain as well as an increased recurrence-free interval.

**Conclusions:**

The microsurgical approach followed by our new protocol described in the study has been beneficial in providing consistent and successful long-term results for these patients.

## Background

Epidermolysis Bullosa (EB) is a group of rare genetic muco-cutaneous diseases characterized by fragile skin and mucosal tissues that blister following minimal trauma. EB may be caused by changes (deleterious mutations) in at least 16 genes mainly involved in maintaining adhesion of the epidermis to the dermal–epidermal junction and underlying dermis [1].

It affects about 20 people out of 1 million born in the US and prevalence is about 11 cases /1,000,000 live births [2]. The clinical diagnosis is confirmed by immunofluorescence antigen mapping and genetic testing [3].

Although 30 subtypes are described, the main clinical types of EB are: simplex (if the blisters are within the basal keratinocytes), junctional (within the lamina lucida), dystrophic (in the superficial papillary dermis), and Kindler syndrome (a mixed type) [1, 4].

Dystrophic EB may be transmitted as an autosomal dominant or recessive subtype.

Recessive dystrophic EB (RDEB) is the more frequent DEB subtype with a prevalence of about 2 in 1,000,000 population [2, 5] and it is related to the loss of collagen VII expression that causes generalized skin blisters at birth, involvement of mucous tissue (gastrointestinal tract), chronic anemia, poor nutrition, recurrent infection, severe renal complication and squamous cell carcinomas [6].

This disease has a devastating impact on the quality of life of patients and requires multi-disciplinary team support [7]. In addition to blisters and the manifestations of skin and mucous membranes, the disease causes several functional deficiencies. In the hands, for example, the repeated minimal trauma, the abnormal inflammatory dysbalance and the fibrotic changes associated with lack of collagen VII lead to pseudosyndactyly with partial and / or complete loss of the interdigital spaces, flexion contractures of the joints and adduction contracture of the thumb [8, 9]. The severe form of contractures is known as “mitten hand” or “cocoon hand” deformity.

Although various gene and protein therapies have been investigated, nowadays there is not cure for EB. The treatment consists of skin care, prevention of skin trauma, adequate nutrition, and medical and surgical treatment if indicated [10].

**Fig. 1:**
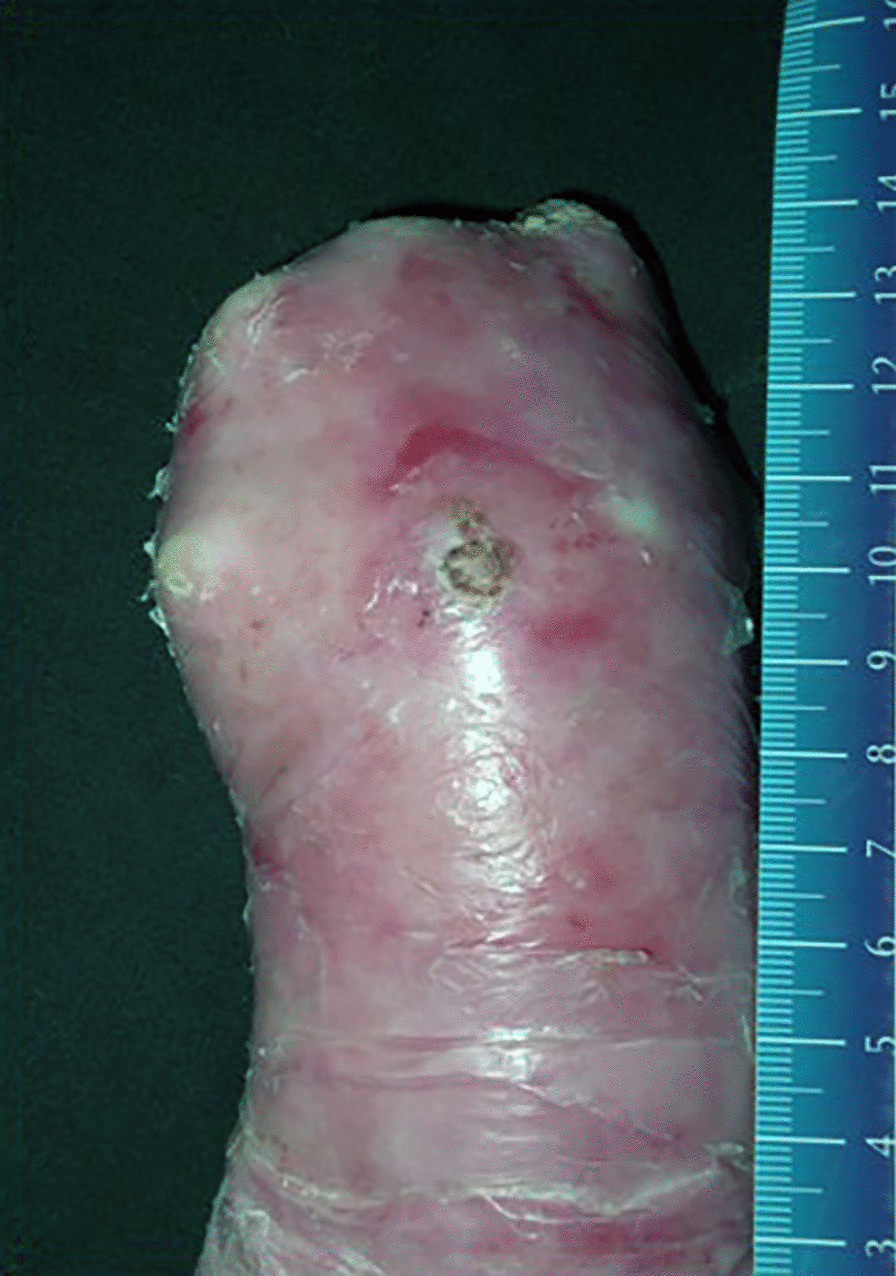
appearance of mitten-hand with imprisonment of the fingers, thumb adduction and extensor deficit

Surgical treatment of these patients is a challenge for the hand surgeon; obtaining successful results and avoiding recurrences are still major problems. In the literature, surgical approaches have been described to correct hand malformations in EB [11–13]. The aim of this treatment is a temporary release, as the pseudosyndactyly of these patients has a high tendency to relapse. Furthermore, the fragility of these patients limits their resilience to anesthesiological procedures. Post-operative treatment is also burdened with very painful dressings and, especially in children, intravenous sedation or a nerve block are often required [14]. In our Department, we have 15 years of experience treating “mitten hands” (Fig. 1) in patients with EB; over the years our surgical and anesthesiological protocol has changed, becoming less invasive and more tolerated by patients. In this manuscript, we describe our protocol combining microsurgery and the use of bilayer dermal regeneration template-gloves for treatment of hand pseudosyndactyly in EB patients. Outcomes in a series of EB patients treated with the new protocol were compared with results of a previous protocol based on surgery followed by dressing with vaseline gauze. The novel protocol proved superior in terms of healing time, hospital stay time, dressing pain, and recurrence-free interval.

## Methods

### Study population

In a retrospective study the Authors collected the data of 24 consecutive patients affected by dystrophic EB who underwent surgical treatment at the Plastic and Reconstructive Surgery Department in Ospedali Riuniti OORR, Foggia, Italy, between January 2001 and January 2016. All patients suffered by severe pseudosyndactylies or mitten hands, with the functional impairment.

The Authors defined 2 groups (A and B). Group A comprised patients treated between 2001 and 2005 who underwent surgical release of the digital rays and dressing with vaseline gauze, while group B included patients treated between 2006 and 2016 with the new protocol combining microsurgery and reconstruction with “gloves” made with INTEGRA^®^ dermal regeneration template.

The aim of this study was to compare our new protocol in the treatment of mitten hand in EB patients with our previous surgical treatment. We have evaluated the following variables: healing time, hospital stay time, discomfort for the patient (through VAS-scale) during dressings, free-recurrence interval.

### First protocol for pseudosyndactyly surgical treatment

From 2001 to 2005, treatment was based on debridement of the pseudosyndactyly and, in mitten hand, temporary arthrodesis in extension of the digital rays with application of K-wires. Coverage of the bloody areas was provided by dressings with vaseline gauze.

**Fig. 2:**
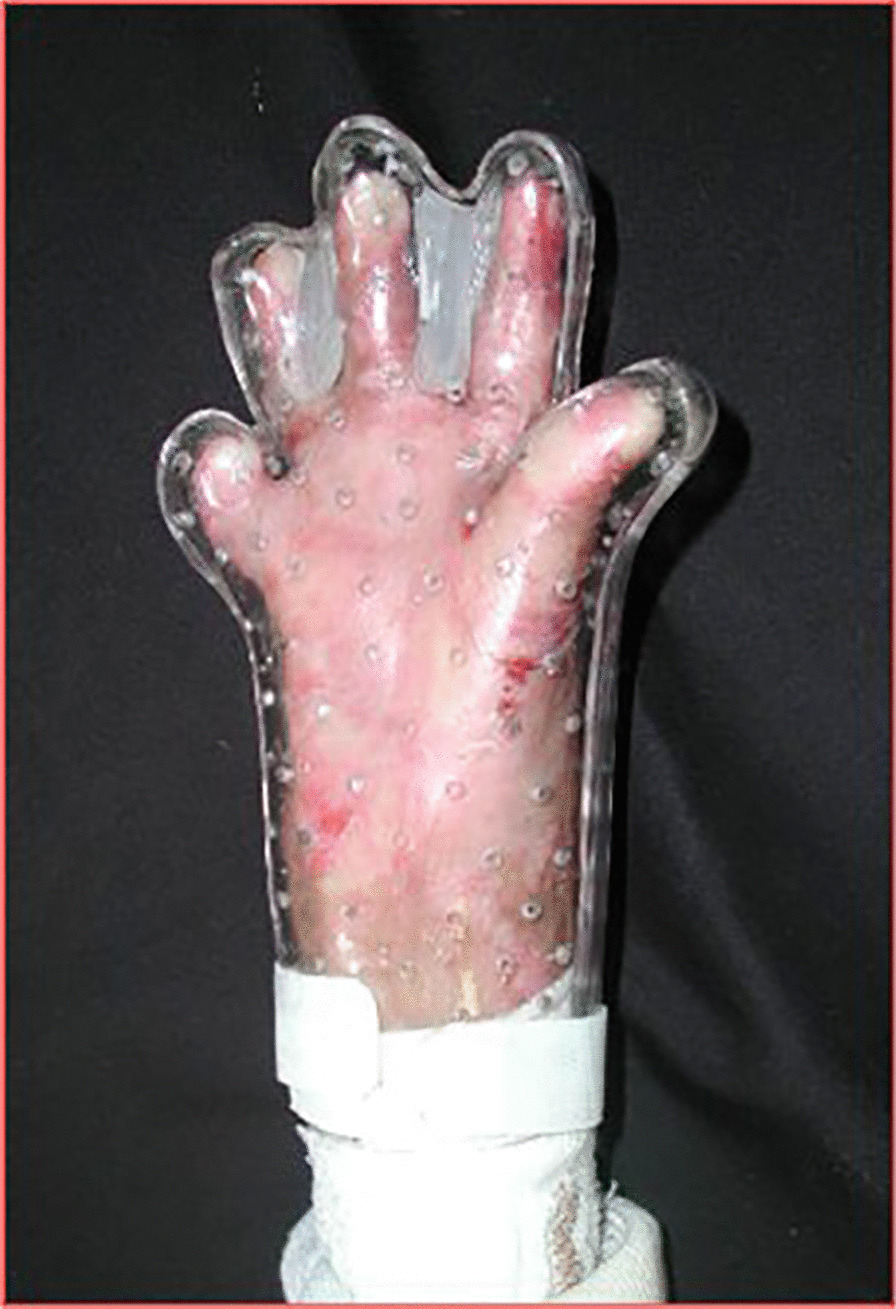
a custom-made splint in perforated thermoplastic material

The patients underwent dressing change every 48–72 h and, in selected cases, given intravenous sedation, until complete epithelialisation. Usually 3 weeks after hand release, the hand therapist fashioned a custom-made splint in perforated thermoplastic material (Fig. 2), and started with functional exercises.

### Innovative protocol for pseudosyndactyly surgical treatment

From 2006 to 2016, the Authors started an innovative protocol called IGP as "INTEGRA^®^-gloves protocol".

The protocol was approved by the Local Medical Ethics Committee in accordance with the Helsinki Declaration of 1975, as amended in 2008. Written informed consent was obtained from parents of all patients for inclusion in this protocol treatment.

The surgical procedure is performed in nerve block anesthesia (using lidocaine 0.5–1% with naropine 1%) and intravenous sedation. Through the use of magnifying loupes (3.5×), the surgeons performed surgical debridement of the pseudosyndactylies more precisely and safely. In fact, the use of microsurgical technique allows us to perform the release of scar contractions with greater preservation of vascular-nerve pedicles which, especially in paediatric patients, are very thin and easily vulnerable.

**Fig. 3:**
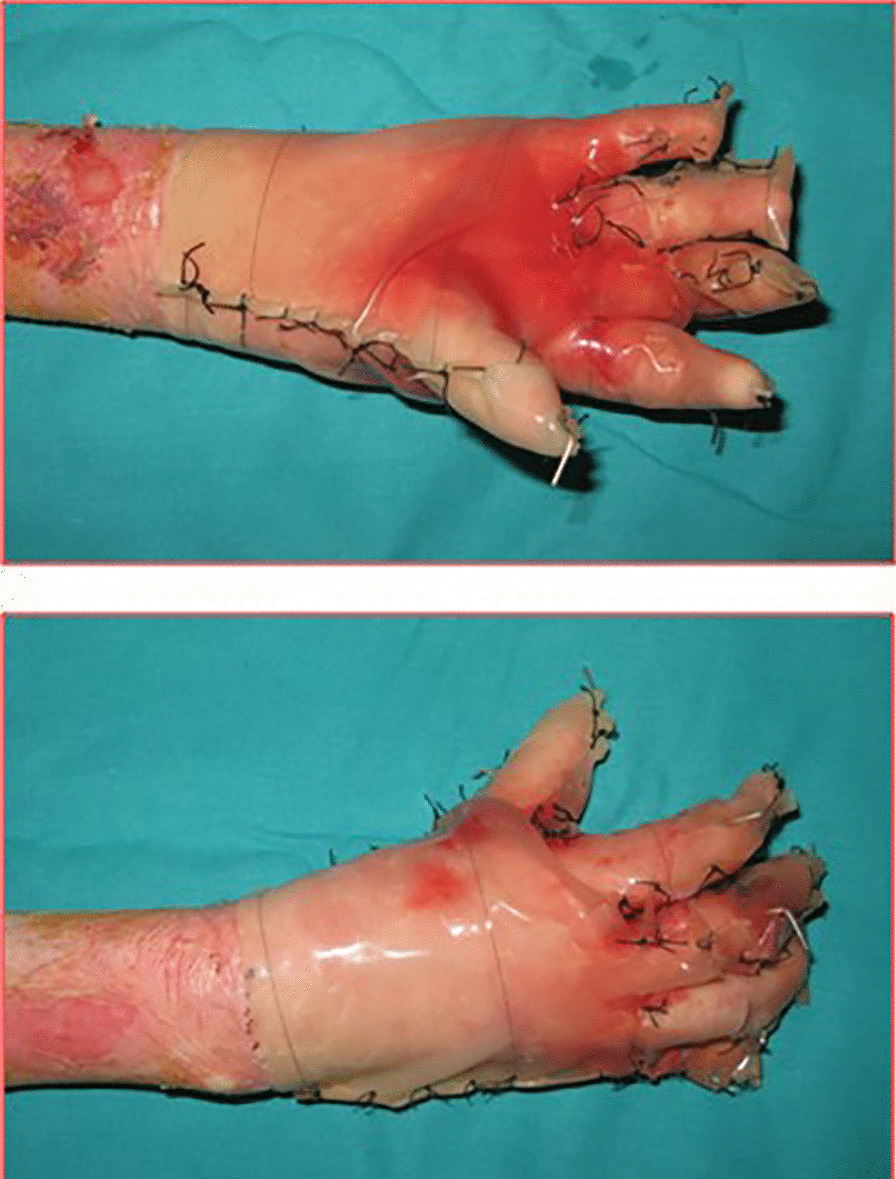
double-layer custom-made gloves with INTEGRA^®^

In all hands K-wires were applied to fix the metacarpo-phalangeal joints in extension, taking care not to induce ischemia in the digital rays. After release, the coverage was performed through a "tailoring" of a double-layer dermal substitute (INTEGRA^®^ dermal regeneration template). These custom-made gloves (Fig. 3) were applied to the hands of patients with absorbable sutures. We used bioengineered tissue on all the surface of the hands, for the known INTEGRA^®^ anti-inflammatory properties. The rationale for this choice was that a reduction in tissue inflammation is very effective in reducing pain during post-operative dressings. Indeed, the presence of the non-adhering silicone layer of the dermal substitute resulted in a painless renewal of dressings.

The first dressing change was performed on the eighth postoperative day, without the need for analgesic sedation or nerve block. Successive dressing changes were performed every 7 days in outpatient department. Around the 30th day, the silicone layer was removed and the few residual areas denuded were medicated with vaseline gauze until complete healing. The use of silicone night splints was recommended for 60 days.

A specific physiotherapy was carried out by experienced hand therapists, followed by home therapy.

### Statistical analyses

We present summary statistics as means with standard deviation (Std). The continuous data were assessed for normality of distribution using a Kolmogorov–Smirnov test that revealed a normal Gaussian distribution. Comparison of the two treatment groups was performed using a Mann–Whitney U test for continuous consequent variables. An expert bio-statistician performed the statistical analysis using Statistical Package for Social Sciences (SPSS version 16.0). A value of p less than 0.05 was considered statistically significant.

## Results

**Fig. 4:**
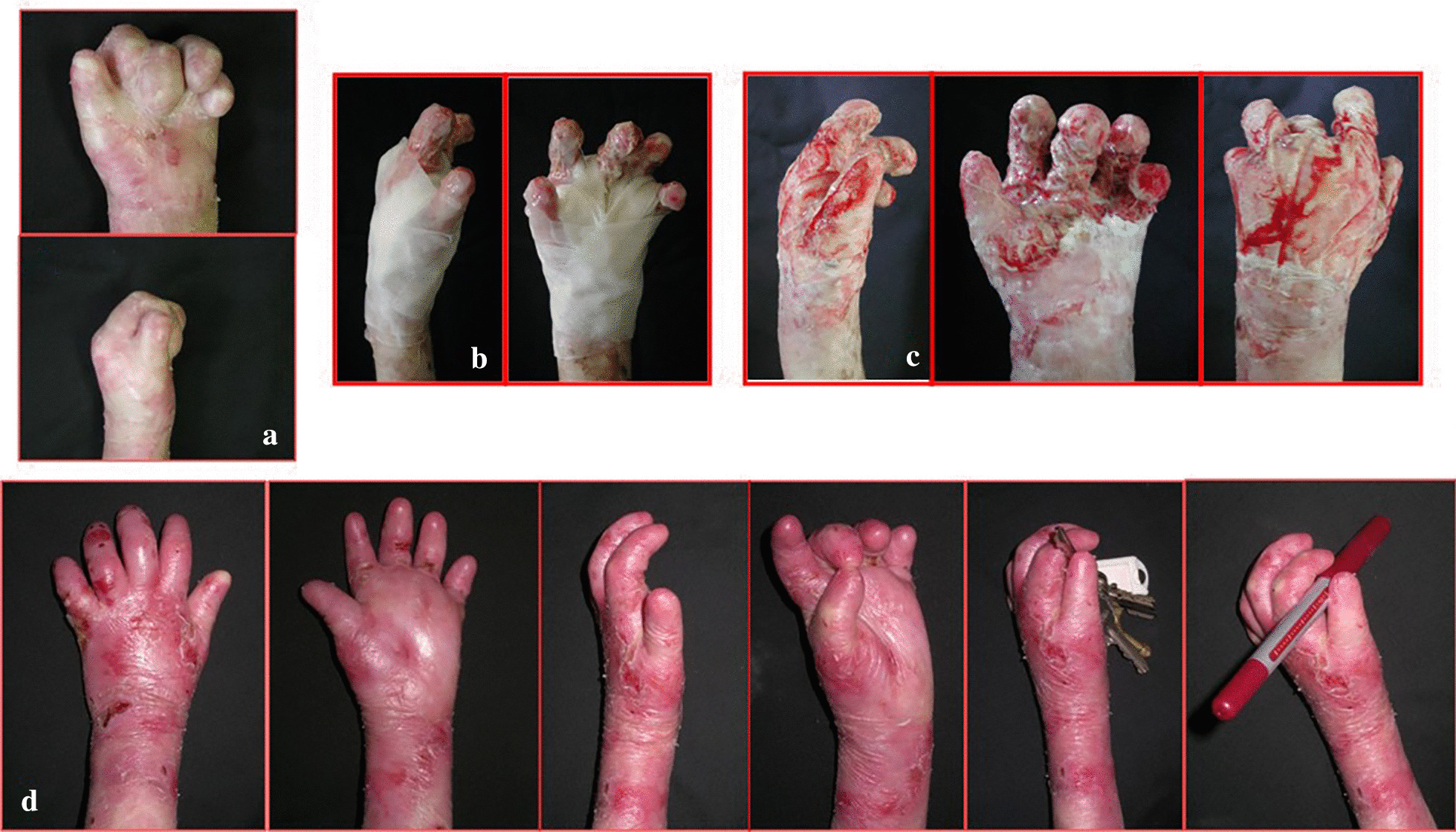
Pre-op (**a**), surgical release of the digital rays and dressing with vaseline gauze (**b**), during healing at 30 days (**c**), follow up at 120 days (**d**)

**Fig. 5:**
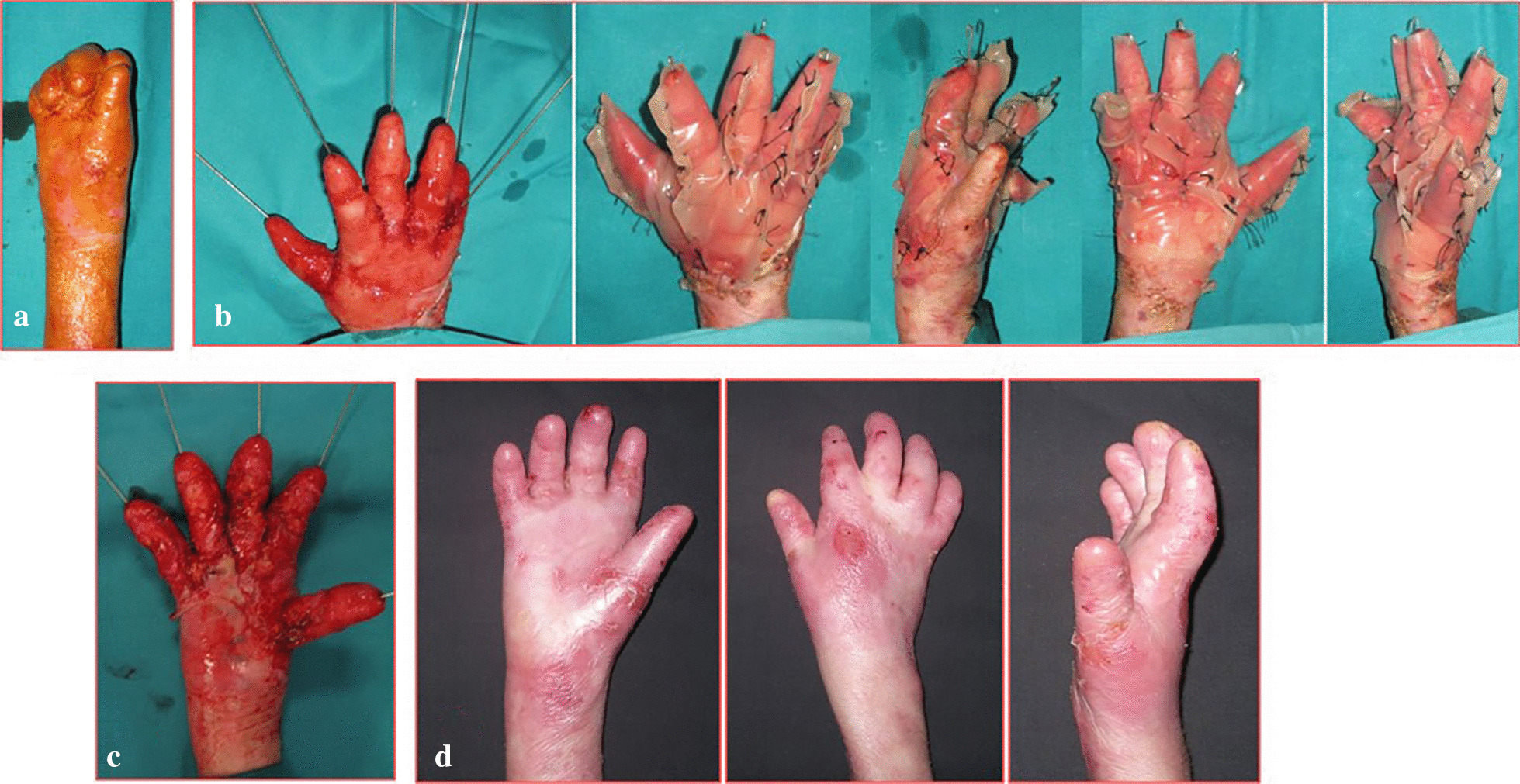
Pre-op (**a**), surgical release of right hand, followed by reconstruction with gloves of INTEGRA^®^ dermal regeneration template (**b**), during healing at 30 days (**c**), follow up at 12 months (**d**)

**Fig. 6:**
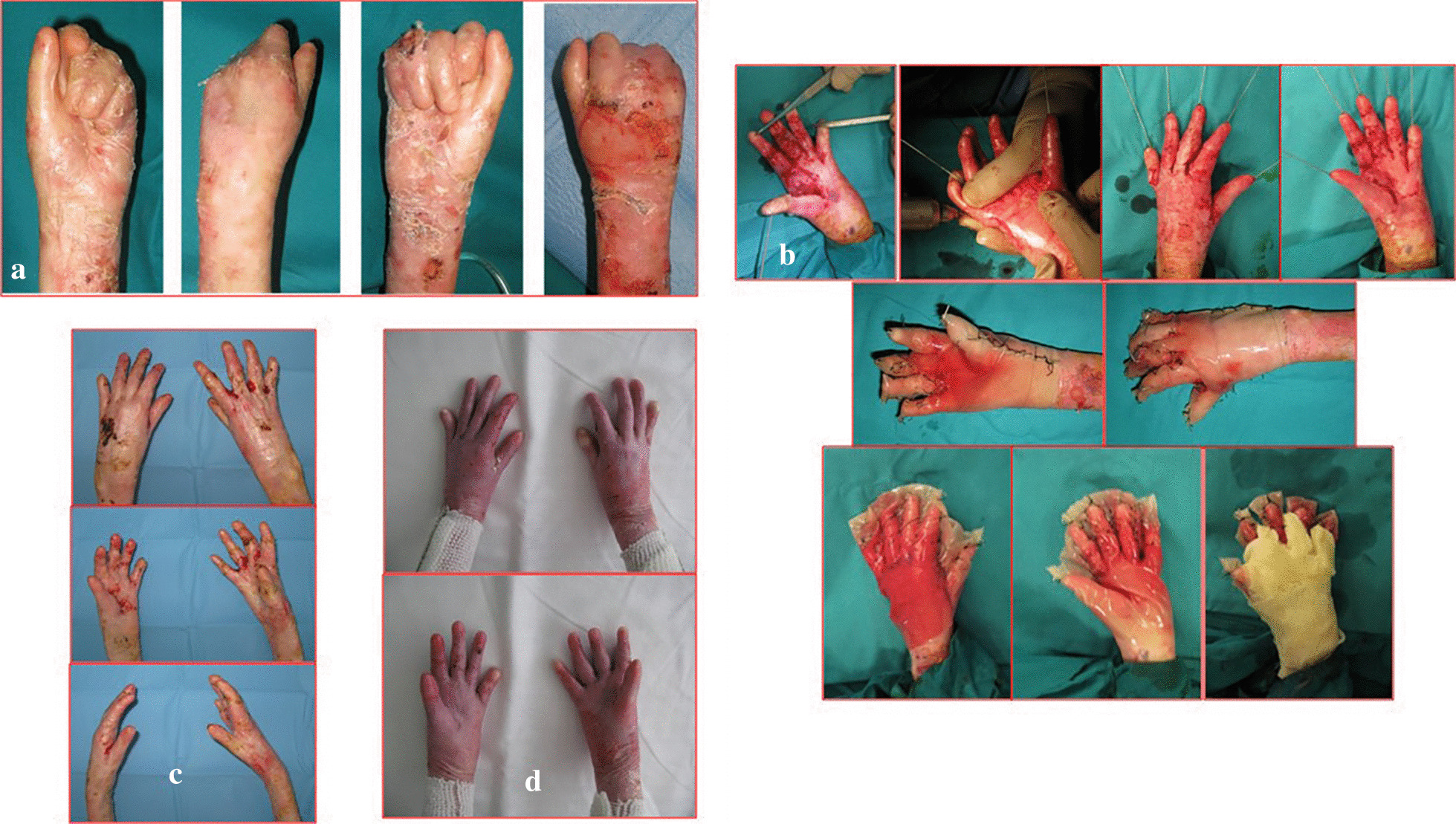
Pre-op (**a**), surgical release of both hands, followed by reconstruction with gloves of INTEGRA^®^ dermal regeneration template (**b**), during healing at 30 days (**c**), follow up at 18 months (**d**)

From January 2001 to January 2016, 24 patients affected by dystrophic EB were treated (13 M, 11F). The range of patient age was 6–15 years old (mean age 8.7). Eleven patients (7 M–4F), with a total of 15 mitten hands, underwent surgical release of the digital rays and dressing with vaseline gauze (Group A) (Fig. 4). Thirteen patients (6 M–7F), with a total of 19 mitten hands, underwent surgical release of the web spaces, finger and wrist contractions, followed by reconstruction with gloves of INTEGRA^®^ dermal regeneration template (Group B) (Figs. 5, 6). The mean age in Group A was 10.1 ± 0.7, and 7.3 ± 2.5 in group B (see Table 1).

**Table 1:** Characteristics of epidermolysis bullosa patients undergoing the two types of surgical approaches for hand pseudosyndactyly

	Group A	Group B
Patients	11	13
M–F	7 M–4F	6 M–7F
Age (mean ± SD)	10.1 ± 0.7	7.3 ± 2.5
Age range	6–13	7–15
Hands	15	19

Group A: Patients underwent surgical release and dressing with vaseline gauze

Group B: Patients underwent surgical release and dressing with Integra^®^

The minimum follow-up was 18 months (range 1.5–9 years).

In both groups treated, no patient reported major complications such as infection and total necrosis of the fingers. In Group A, the average hospital stay time was 29.6 ± 1.6 days (range 16–45 days), while in Group B it was 3.6 ± 0.8 days (range 2–5 days), with a *p*-value = 0.021.

Complete healing times (with reepithelialization of more than 90% of the hand) were 46.4 ± 3.4 days for Group A and 24.6 ± 2.9 days for the group treated with INTEGRA^®^, with a *p*-value = 0.008.

In group A, patients had discomfort after dressings, with an average value of 7.6 on the VAS scale, whereas B group scored an average of 0.6, with a *p*-value = 0.034.

Dressings were painful for the patients of Group A, in particular when, to completely release their fingers, the vascular-nerves pedicles were exposed. Patients also needed for a sedation or nerve block. In order to carry out these procedures, it was necessary to have an average hospital stay of about 30 days with great difficulties for the patient and the family members.

The average time to recurrence was 19.1 ± 5.3 months for group A and 31.1 ± 4.7 months for B, with a *p*-value = 0.045 (Table 2).

**Table 2:** Outcomes of the two types of surgical approaches for hand pseudosyndactyly

	Group A	Group B	*p*-value
Healing times (days, mean ± SD)	46.4 ± 3.4	24.6 ± 2.9	0.008
Hospital stay time (days, mean ± SD)	29.6 ± 1.6	3.6 ± 0.8	0.021
Discomfort for the patient (through VAS-scale, mean)	7.6	0.6	0.034
Free-recurrence interval (months, mean ± SD)	19.1 ± 5.3	31.1 ± 4.7	0.045
Follow-up range (years, range)	3.7–8	1.5–9	–
Major complications	0	0	–

Group A: Patients underwent surgical release and dressing with vaseline gauze

Group B: Patients underwent surgical release and dressing with Integra^®^

Thus, there was a statistical significant difference in favour of group B in hospital stay, complete healing times, discomfort during dressings, and recurrence-free time.

## Discussion

Recessive dystrophic forms of EB are burdened with a high psychological impact for patients and their families. A multidisciplinary approach to manage the disease is fundamental and surgery plays a critical role. “Mitten hands” compromise the functional development of the hands in children with EB. So, an early surgical approach is of paramount importance. Fine et al. showed that about 61% of recessive dystrophic EB patients require hand surgery, typically needing 5 or more operations [5].

Pseudosyndactylies in patients with this disease are characterized by high rates of recurrence [7, 9, 11–13, 15].

Surgical challenges include impaired wound healing and risk of iatrogenic trauma. In fact, the scar tissue of the hands can alter the anatomical structures, including the vascular-nerve pedicles; so minimal inadvertence during surgical procedures can cause local ischemia and subsequent partial or total skin necrosis.

Nevertheless, surgery is crucial to correct severe hand deformities, improving patient’s quality of life. In a study of 1995, Ciccarelli et al. [15] proposed these indications for surgery: palmar contracture, contracture of the proximal interphalangeal (IP) joint > 30°, severe small finger deformity, pseudosyndactyly extending to the proximal IP joint, severe impairment of daily activities.

Conventional surgical techniques for “mitten hand” are limited to the release of pseudosyndactylies and wound coverage, achieving optimal epithelialisation, beginning early mobilization, and providing long-term stability with minimal recurrence [7]. Various methods are described, but no consensus exists.

Healing by secondary intention is described [12], but larger wounds require coverage to avoid infection, delayed healing with fibrosis and recurrence of contractures. In these cases, the use of skin graft (split-thickness or full-thickness), cultured keratinocytes, and cellular allograft dermal matrix (such as Apligraf) or acellular biosynthetic material (such as Biobrane or AlloDerm-GBR) has been described in the literature [7, 16–26].

Split-thickness skin grafts (STSG) provide coverage of wide areas, however, availability of healthy skin for autologous grafting and difficult healing at the donor site can be major problems in EB patients. Moreover, split-thickness skin graft usually provides unstable coverage with high risk of early recurrences [7, 11].

Full-thickness skin graft is useful in the first web space and delays recurrence of contracture as compared with STSG, but the donor sites are very limited.

The use of skin substitutes in patients with EB has been described in the literature; often in combination with skin grafts.

Recent studies focused on allogenic skin substitutes derived from human keratinocytes and fibroblasts. In particular, the studies of Fivenson [19] and Falabella [22], in the early 2000’s, demonstrated the successful results in the treatment of severe hand deformities of EB patients, using of an allogenic bilayer cellular skin equivalent called Apligraft^®^. Other studies analyzed the utility of other materials such as autologous and allogenic epidermal keratinocyte grafts, amniotic membrane grafts, and acellular dermal allograft [20, 21, 24, 25]. Although some disadvantages are described such as need autologous STSG harvesting, prolonged time healing, and high costs [20, 25], nevertheless the new dermal support created by the acellular dermal matrix can prevent recurrence of the pseudosyndactylies in the long term [26].

Hand function preservation and the time interval to relapse are the most important parameters in evaluating surgery efficacy.

Following a careful analysis of our surgical procedures, we were able to improve outcomes in our patients by combining the use of microsurgery with the application of Integra ^®^ dermal regeneration template.

In fact, we believe that magnifying loupes have allowed us to preserve the vascular-nervous pedicles in these patients, especially if in paediatric age. The higher skills and expertise of the Surgeons using the microscopic lenses in the release of pseudosyndactylies also helped to gain better results avoiding surgical damages.

We believe that through our protocol using a bilayer dermal regeneration template it is possible to avoid skin grafting and therefore iatrogenic damage and further surgery.

Artificial dermis, as Integra^®^, is an acellular purely bilaminate synthetic construct consisting of an outer silicone (polysiloxane) semipermeable membrane and an inner porous matrix of collagen-glycosaminoglycan.

The outer layer serves as an epidermal substitute and provides mechanical protection, and heat and moisture modulation of the wound, prevents formation of wound granulation tissue and increases tear strength of the custom-made gloves.

On the other hand, the inner layer, composed of bovine tendon type I collagen cross-linked to chondroitin-6-sulfate, is a biologic-based acellular dermal scaffold and promotes cellular ingrowth (it is histioconductive/histioinductive and allows for fibroblast proliferation and migration into the dermal scaffold and capillary growth). In particular glycosaminoglycan provides elasticity to the matrix, controls the biodegradation rate, and maintains a more open pore structure that allows cell migration into the matrix.

The porous layer of the matrix is strictly applied to the wound bed at the first stage. It acts as a template for the ingrowth of host fibroblasts and endothelial cells and is gradually replaced by host (endogenous) collagen, forming a new dermal layer (neodermis).

Integra^®^ dermal regeneration template has been originally designed for treatment of full thickness skin lesions in a two-step procedure, where the second step consists of peeling off the silicone layer and applying an autologous split-thickness skin graft on the neodermis [27, 28]. Importantly, in our protocol for EB hands we use the Integra^®^-glove as an advanced wound dressing avoiding the second phase of skin graft and obtaining an almost complete and spontaneous re-epithelialization.

On the other hand, the use of artificial dermis offers many advantages, such as: immediate availability, possibility to cover large defects, no donor-site morbidity, good scarring, and early recovery.

In addition, the anti-inflammatory properties and physical characteristics of INTEGRA^®^ allowed us to perform pain-free dressing and achieve more stable coverage over time. The coverage of noble structures, such as vascular and nerve pedicles, has been found to perform well. We believe that coverage through our technique has allowed a lower rate of early recurrence. In fact, inter-digital spaces have had a reduced tendency toward fusion during the healing phase, probably thanks to the INTEGRA^®^ layer. The severity of the primary disease, the degree of hand deformity and the age of the patients at their initial referral are also relevant factors, which need to be addressed in future studies.

Due to their reduced invasiveness, these surgical procedures can be carried out in local anaesthesia and sedation; in particular, we performed: regional anaesthesia (brachial plexus), or deep sedation with fentanyl and propofol plus local anaesthetic infiltration, or deep sedation with ketamine and fentanyl plus local anaesthetic infiltration [29]. We avoid to perform general anaesthesia because of high risk of iatrogenic damage of intubation in this kind of patients. With our new protocol the medications are painless, so we don’t use anaesthesia.

## Conclusions

The proposed protocol is proven safe, repeatable, and simple to execute. Patients reported less discomfort after Integra^®^-gloving than after the previous treatment option, and we believe that every effort to reduce patients’ distress and shorten the number of hospital visits is justified. The high rate of recurrence after treatment of “mitten hands” necessitates a lasting approach that offers stable coverage with less discomfort for patients. The limitations in our study are: its small simple size and a method for assessing wound healing that is based on the surgeon’s experience and qualitative rather than standardized. Moreover, it doesn’t compare the results of different dermal substitutes; at last, there is not preoperative evaluation of pseudosyndactylies using a validated scale.

In spite of these limitations, the significant differences between both groups cannot be ignored, suggesting the clear repercussions of this new protocol on long lasting healing and on quality of life (QoL) of EB patients.

The results of this relatively small study (34 hands) have led to an adaptation of the EB-hands treatment at our Centre; we are consequently collecting and analyzing other data in order to expand this study in future and produce a “stronger” analysis.

The authors are convinced that the findings in this study may be useful for other surgery Units. We believe that the use of this dermal regeneration template in dystrophic EB hand deformities should be included in the routine surgical treatment protocol.

However, it would be desirable to have a multi-center study with other Hand Surgery departments that take care of these deformities.

Finally, a future study should also measure with validated instruments the impact of pseudosyndactyly surgical treatment on quality of life of EB patients, as QoL represents a primary and essential outcome of any treatment strategy for this extremely disabling disease manifestation.

## Data Availability

The datasets used and/or analyzed during the current study are available from the corresponding author on reasonable request.

## Abbreviations

EB: Epidermolysis Bullosa
F: Female
IGP: INTEGRA^®^-gloves protocol
IP: Interphalangeal
M: Male
QoL: Quality of life
RDEB: Recessive Dystrophic Epidermolysis Bullosa
SPSS: Statistical Package for Social Sciences
Std: Standard deviation
STSG: Split-thickness skin grafts
VAS: Visual analogue scale
